# Diversity and Functional Predictions of Gut Microbiota in Vietnamese Children Aged 6–24 Months with Persistent Diarrhea of Unknown Etiology

**DOI:** 10.3390/pathogens14111136

**Published:** 2025-11-09

**Authors:** Thi Thanh Nga Pham, Trong Khoa Dao, Thi Viet Ha Nguyen, Thi Bich Thuy Phung, Hong Duong Nguyen, Thi Quy Nguyen, Thi Thu Hong Le, Thi Huyen Do

**Affiliations:** 1 Vietnam National Children’s Hospital, 879 La Thanh Street, Hanoi 11528, Vietnam; phamthithanhnga1986@gmail.com (T.T.N.P.); vietha@hmu.edu.vn (T.V.H.N.); thuyphung.nhp@gmail.com (T.B.T.P.); 2 Department of Pediatrics, Hanoi Medical University, 1 Ton That Tung Street, Hanoi 11520, Vietnam; 3 Institute of Biology, Vietnam Academy of Science and Technology (VAST), 18 Hoang Quoc Viet Street, Hanoi 11307, Vietnam; 4 School of Biotechnology, Graduate University of Science and Technology, Vietnam Academy of Science and Technology (VAST), 18 Hoang Quoc Viet Street, Hanoi 11307, Vietnam

**Keywords:** 16S rRNA sequencing, children, dysbiosis, functional prediction, gut microbiota, persistent diarrhea

## Abstract

Persistent diarrhea remains a significant cause of morbidity in young children, yet the role of gut microbiota has not been fully clarified. This prospective study evaluated the diversity and predicted functions of the gut microbiota in 30 children aged 6–24 months with persistent diarrhea of unknown etiology (patient group, PG) and 30 healthy controls (healthy group, HG). Nearly full-length 16S rRNA genes from fecal bacterial metagenomic DNA were sequenced and taxonomically annotated. Subsequently, all downstream analyses, including diversity assessment, differential abundance and functional prediction analyses, and data visualization, were performed using R software (version 4.5.0, 2025). The PG showed lower Shannon and higher Simpson indices than the HG (*p* < 0.05), reflecting reduced microbial diversity. At the phylum level, Firmicutes predominated in the PG, whereas Actinobacteriota, Bacteroidota, and Verrucomicrobiota were more abundant in the HG (|log_2_FC| > 1 and FDR < 0.05). At the genus and species levels, the PG exhibited a marked depletion of essential commensals such as *Bifidobacterium longum*, *Faecalibacterium*, *Lactobacillus*, and *Eubacterium*, alongside an enrichment of opportunistic taxa including *Klebsiella*, *Enterococcus lactis*, and *Streptococcus* spp. (FDR < 0.05). Functional predictions using PICRUSt2 indicated an enrichment of carbohydrate metabolism and reductions in amino acid metabolism, B-vitamin pathways, and the biosynthesis of endogenous antibiotics (FDR < 0.05). These findings suggest that the PG harbors a dysbiotic gut microbiota characterized by reduced diversity, depletion of key commensal taxa, expansion of opportunistic bacteria, and potentially adverse shifts in metabolic functions.

## 1. Introduction

Diarrhea remains a major cause of morbidity and mortality in children under five years of age worldwide, with the highest burden reported in South Asia and sub-Saharan Africa [1,2]. In Vietnam, the incidence among children under five remains high, with an estimated 0.81 episodes per child per year [3].

According to the World Health Organization (WHO), persistent diarrhea is defined as the passage of more than three loose or watery stools per day, usually of acute onset and lasting longer than 14 days, most commonly caused by bacterial, viral, or parasitic pathogens [1]. Diarrhea persisting beyond four weeks is classified as chronic diarrhea [4,5], with etiologies not limited to gastrointestinal infections but also encompassing food allergies, intolerances, and inflammatory bowel disease [6,7]. Current evidence suggests that persistent diarrhea accounts for approximately 5–18% of all diarrheal episodes in children [8], with those aged 6–24 months identified as the most vulnerable group [9].

The identification of infectious etiologies still predominantly relies on conventional methods such as Gram staining, microscopic examination, culture, toxin assays, and antigen–antibody tests [10]. Although culture was previously considered the gold standard for bacterial identification, it is now recognized that approximately 60–85% of species cannot survive or proliferate under routine culture conditions [11]. While many of these uncultivable species may have limited clinical relevance, the limitation of culture-based method still hampers the detection of certain fastidious or clinically significant microorganisms involved in gastrointestinal infections. Advances in molecular diagnostics, particularly PCR, have provided more sensitive approaches for pathogen detection. Nevertheless, the detection of DNA or RNA at very low concentrations is not always clinically meaningful, as the presence of genetic material does not necessarily indicate viable or pathogenic microorganisms [12]. Consequently, determining the microbial cause of diarrhea remains challenging, even when multiple diagnostic modalities are employed, with up to 40% of cases failing to identify a specific pathogen [13]. Furthermore, in regions with high rates of asymptomatic intestinal carriage, such as many developing countries, the identification of microorganisms in children with persistent diarrhea does not always imply causality [12,14,15].

This poses a considerable challenge in clinical practice, emphasizing the necessity for innovative diagnostic approaches and a more comprehensive understanding of disease pathogenesis, particularly concerning the interplay between persistent infections and gut microbiota dysbiosis [16]. In healthy individuals, the gut microbiota is generally stable and maintains a balanced ecosystem, particularly within the core bacterial community [17,18]. Dysbiosis refers to a disruption in microbial taxa’s composition or relative abundance compared with healthy individuals’ microbiota [19]. Several studies on children with diarrhea have shown changes in the gut microbiota, characterized by decreased diversity and richness, an increased abundance of opportunistic bacteria, and a concurrent decline in beneficial anaerobes and commensal taxa [20,21,22,23].

Culture-independent methods, particularly metagenomic approaches, have been developed to directly analyze microbial genetic material (DNA/RNA). Among these, 16S rRNA gene sequencing is widely used to classify bacteria based on the hypervariable regions of the 16S rRNA gene, providing critical insights into community structure and diversity and detecting fastidious organisms and unculturable taxa [10,24]. Although 16S rRNA sequencing does not provide direct functional data, predictive tools such as PICRUSt2 allow inference of functional potential, thereby enhancing our understanding of microbiota contributions to persistent diarrhea [25].

In Vietnam, data on the gut microbiota of children aged 6–24 months with persistent diarrhea of unknown etiology are currently lacking. To date, no study has comprehensively examined microbial diversity, community composition, and inferred functional potential in this population. Therefore, this study aimed to comprehensively characterize the gut microbiota in affected children, thereby providing a scientific basis for improved diagnosis and potential clinical interventions.

## 2. Materials and Methods

### 2.1. Study Design and Participants

A prospective descriptive study was conducted at the Vietnam National Children’s Hospital from July 2023 to December 2024, enrolling 60 children aged 6–24 months, including 30 healthy controls (Healthy Group, HG) and 30 patients with persistent diarrhea of unknown etiology (Patient Group, PG).

The HG comprised healthy children without diarrhea, free from acute or chronic infections, with no antibiotic exposure for at least one month, and no history of diarrhea within at least three months prior to sampling. All HG participants tested negative for 24 common diarrheal pathogens using multiplex PCR assay (QIAstat-Dx Gastrointestinal Panel, Qiagen, Hilden, Germany). The PG included children with acute-onset diarrhea, defined as three or more loose stools per day persisting for 14–28 days. Stool microscopy revealed evidence of infection, indicated by the presence of at least one leukocyte or erythrocyte per 5–10 high-power fields, while both stool culture and multiplex PCR results were negative. Children with other acute or chronic diseases unrelated to diarrhea or a history of gastrointestinal surgery were excluded.

The study was approved by the Ethics Committee of the Vietnam National Children’s Hospital (Approval No. 1875/BVNTW-HĐĐĐ, dated 10 July 2023), and written informed consent was obtained from the parents or legal guardians of all participants.

### 2.2. Sample Collection, Processing, and DNA Extraction

Fecal samples were collected individually in accordance with the Handbook for Use of Testing Services (ST.XN.4.4), No. 13/CT-BVNTW, issued on 22 March 2023, by the Vietnam National Children’s Hospital. The samples were transported to the laboratory within 60 min of collection. At the Department of Microbiology, specimens underwent direct microscopic examination, followed by stool culture to detect common enteric bacterial pathogens, including toxigenic *Escherichia coli*, *Shigella* spp., and *Salmonella* spp. For molecular analysis, fecal specimens were stored at 2–8 °C for a maximum of 72 h and subsequently analyzed using the QIAstat-Dx^®^ Gastrointestinal Panel (Qiagen, Hilden, Germany) at the Department of Molecular Biology of Infectious Diseases. This multiplex real-time PCR can detect 24 common pathogens, including 4 parasites (*Entamoeba histolytica*, *Cryptosporidium* spp., *Giardia lamblia* and *Cyclospora cayetanensis*), 14 bacteria (*Vibrio vulnificus*, *V. parahaemolyticus*, *V. cholerae*, *Campylobacter* spp., *Salmonella* spp., *Clostridioides difficile* (*tcd*A/*tcd*B), *Yersinia enterocolitica*, Enteroaggregative *E. coli* (EAEC), Enterotoxigenic *E. coli* (ETEC), Shiga toxin–producing *E. coli* (STEC), Shiga toxin-producing *E. coli* serotype O157:H7, Enteropathogenic *E. coli* (EPEC), Enteroinvasive *E. coli* (EIEC)/*Shigella* and *Plesiomonas shigelloides*), and 6 viruses (*human adenovirus* F40/F41, *Norovirus* GI and GII, *Rotavirus* A, *Astrovirus* and *Sapovirus* GI, GII, GIV, GV).

Following the initial analyses, from 51 healthy children and 67 children with persistent diarrhea, the remaining 30 stool samples negative for common diarrheal pathogens from each group (HG and PG) were stored at −80 °C until bacterial separation followed by DNA extraction at Department of Omic Technologies and Applications, Biology Institute. Bacterial DNA was extracted using the GeneJET Genomic DNA Purification Kit (Thermo Scientific, Waltham, MA, USA) in accordance with the manufacturer’s instructions. Additionally, metagenomic DNA was extracted using the phenol–chloroform method described in a previous study [26]. The DNA pellet was dissolved in 50 µL of TE buffer and subsequently analyzed by electrophoresis on a 0.8% agarose gel. DNA quality and concentration were further assessed using a NanoDrop NC-2000C spectrophotometer (Implen, Isogen, PW De Meern, The Netherlands). Equal amounts of DNA extracted by the two methods from each sample were pooled together to form one. A total of 30 metagenomic DNA samples from PG (designated as PG1–PG30) were subjected to next-generation sequencing (NGS).

By contrast, HG samples contained a consistently fingerprinting types of bacterial diversity and were therefore pooled into 10 samples (designated as HG1–HG10, corresponding to the HV1-HV10 in a previous study [27]). In brief, the pooling was guided by analysis of bacterial diversity using PCR-RFLP, examining 16S rRNA genes digested with *Mbo* I, and was accompanied by a heatmap analysis that clustered samples with similar banding profiles [26]. Within each cluster, equal amounts of DNA from individual samples were combined. The pooled DNA was subsequently re-evaluated for quality and concentration to ensure suitability for sequencing. A total of 10 pooled metagenomic DNA samples from HG were subjected to NGS.

### 2.3. 16S rRNA Gene Sequencing and Bioinformatics Analysis

The nearly full 16S rRNA genes were amplified from 30 ng of template DNA using degenerated primers 27FD: 5′-AGR GTT YGA TYM TGG CTC AG-3′ and 1492RD: 5′-RGY TAC CTT GTT ACG ACT T-3′, followed by purification and library preparation. Library quality was evaluated with the Agilent 2100 Bioanalyzer (Agilent Technologies, Santa Clara, CA, USA) prior to sequencing on the DNBSEQ platform (MGI Tech, Shenzhen, 518083, China) yielding raw Hifi reads. The raw reads were quality-filtered to remove low-quality sequences (Phred score < 20 over a 25 bp sliding window, retained length < 75% after trimming), adapter-contaminated reads, reads containing ambiguous bases (N), and low-complexity reads (≥10 consecutive identical bases). High-quality paired-end reads were then merged into consensus sequences using FLASH (v1.2.11), with a minimum overlap of 15 bp and a maximum mismatch rate of 0.1 in the overlapping region. Finally, the clean data for PG totaled 2.8 Gb, comprising 60,277 to 64,975 Hifi reads per PG sample. The clean data for HG amounted to 2.2 Gb, consisting of 123,417 to 188,140 Hifi reads per HG sample. The read length ranged from 1452 to 1483 bps, and the sequencing quality, assessed by Q30 values, ranged from 91.2 to 92.0%.

Clean sequences were clustered into operational taxonomic units (OTUs) at a 97% similarity threshold using UPARSE (USEARCH v7.0.1090). Chimeric sequences were detected and removed with UCHIME (v4.2.40) against the Gold database (v20110519). Representative sequences from each OTU were retained, and the remaining reads were mapped back using USEARCH GLOBAL to generate an OTU abundance table. Taxonomic annotation was performed with the RDP (Ribosomal Database Project) Classifier (v2.2, confidence threshold 0.6) against the Greengenes (V202210), RDP (Release19 2023-07-20) and Silva (V138 2019-12-16) databases. OTUs that could not be assigned taxonomically or were irrelevant to the study objectives were excluded.

Alpha diversity and taxonomic composition analyses were conducted based on the OTU abundance table and RDP-derived annotations to characterize community structure and the relative abundance of gut microbiota from the phylum to species level. Functional profiling of microbial communities was predicted using PICRUSt2 (v2.3.0-b), which infers Kyoto Encyclopedia of Genes and Genomes (KEGG) Orthologs and metabolic pathways, generating a functional abundance table for further analysis.

### 2.4. Statistical Analysis

All statistical analyses were performed using R software (version 4.5.0, 2025) [28]. Because the HG samples were pooled, statistical analyses were performed in an exploratory manner, and the resulting differences were regarded as descriptive trends.

Differences in alpha diversity indices were assessed using the Wilcoxon rank-sum test (*p* < 0.05). Predicted functional profiles generated by PICRUSt2 were evaluated using the Wilcoxon rank-sum test with *p*-values adjusted by the Benjamini–Hochberg method to control the false discovery rate (FDR). An FDR < 0.05 was used as the reporting threshold for functional analyses. Functional profiles were predicted using PICRUSt2, which provides inferred metabolic potential based on 16S rRNA gene data rather than direct measurements of gene expression or enzymatic activity.

Relative abundance data of taxa at the phylum, genus, and species levels were analyzed to compare gut microbiota composition between the PG and HG. For taxa detected in both groups, differences in relative abundance were evaluated using the Wilcoxon rank-sum test with FDR-adjusted *p*-values, and log_2_ fold change (log_2_FC) values were calculated based on mean relative abundance to assess biological trends. Taxa with |log_2_FC| > 1 and FDR < 0.05 were reported as exploratory biological differences. Fisher’s exact test was applied to presence/absence data with the same FDR adjustment for taxa exclusive to one group. Taxa meeting the FDR < 0.05 threshold in this test were interpreted as showing exploratory differences in occurrence frequency between PG and HG.

## 3. Results

### 3.1. Characteristics of Study Participants

Table 1 summarizes the baseline characteristics of the study participants. Children in the PG had a significantly lower mean age than those in the HG (7.2 ± 2.0 vs. 11.4 ± 3.8 months, *p* < 0.001), with the majority aged 6–12 months (96.7% vs. 63.3%, *p* = 0.002). No statistically significant differences were observed between groups regarding sex, residence, mode of delivery, feeding type (0–4 months), timing of complementary feeding, drinking water source, childcare, or nutritional status (all *p* > 0.05). Notably, 86.7% of children in the PG had used antibiotics within the past 30 days.

### 3.2. Alpha Diversity of the Gut Microbiota

**Figure 1:**
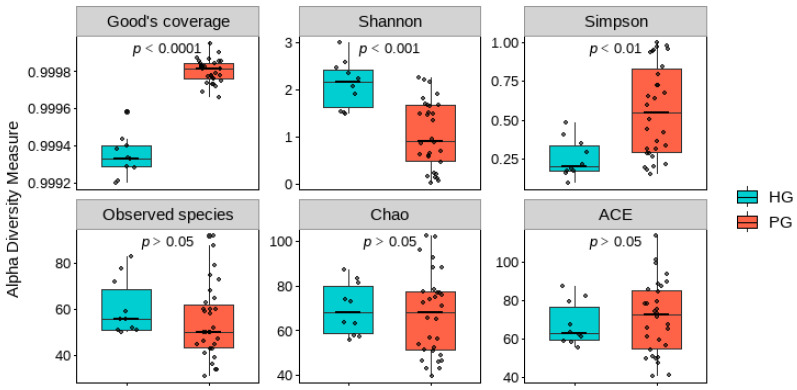
Comparison of six alpha diversity indices between PG and HG. Boxes represent the interquartile range (IQR) from the first to the third quartile, with the black line indicating the median. Whiskers extend to the minimum and maximum values within 1.5 × IQR.

Six α-diversity indices were analyzed, including Good’s coverage, Shannon, Simpson, Observed species, Chao1, and ACE (Figure 1). Good’s coverage exceeded 99.9% in both groups, indicating excellent sequence coverage and adequate sequencing depth. Community diversity and evenness, as measured by the Shannon and Simpson indices, showed differences between PG and HG (Wilcoxon rank-sum test, *p* < 0.05). The Shannon index was higher in HG (median = 2.15, IQR = 0.80) than in PG (median = 0.92, IQR = 1.18), with *p* < 0.001. In contrast, the Simpson index was higher in PG (median = 0.55, IQR = 0.53) than in HG (median = 0.20, IQR = 0.16), with *p* < 0.01. The remaining three indices, which assess species richness (Observed species, Chao1, and ACE), did not show differences between the two groups (*p* > 0.05).

### 3.3. Gut Microbiota Composition

#### 3.3.1. Analysis of Gut Microbiota at the Phylum Level

**Figure 2:**
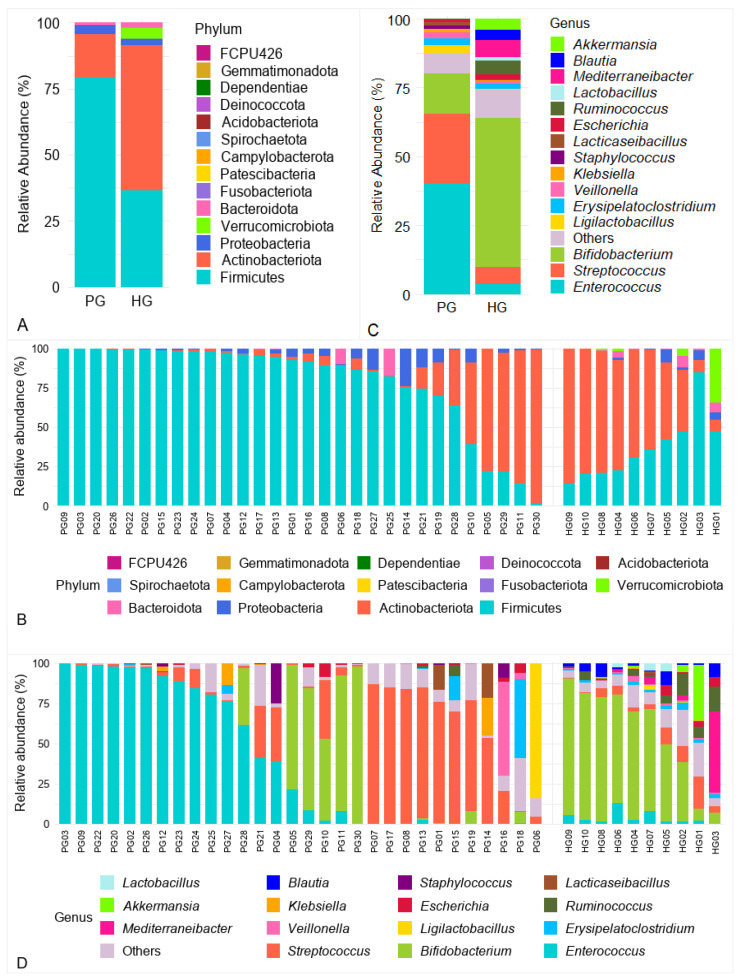
Gut microbiota composition at the phylum and genus levels. (**A**) Relative abundance of bacterial phyla in PG and HG. (**B**) Relative abundance of bacterial phyla in each sample. (**C**) Relative abundance of bacterial genera in PG and HG. (**D**) Relative abundance of bacterial genera in each sample.

A total of 14 bacterial phyla were identified across fecal samples from both groups. Among these, the four predominant phyla were Firmicutes, Actinobacteriota, Proteobacteria, and Bacteroidota, consistently present in the majority of samples from both PG and HG (Figure 2A,B). Firmicutes were markedly enriched in PG, with a mean relative abundance of 79.06 ± 29.13%, higher than in HG (36.47 ± 20.58%; log_2_FC = +1.12; FDR = 0.002). Conversely, Actinobacteriota predominated in HG (54.93 ± 28.32%) but were decreased in PG (16.48 ± 29.51%; log_2_FC = −1.74; FDR = 0.002). Bacteroidota also demonstrated a higher mean abundance in HG (2.05 ± 2.83%) than in PG (0.97 ± 3.56%; log_2_FC = −1.08; FDR = 0.046). Among the phyla exclusively detected in one of the two groups, Verrucomicrobiota was the only phylum showing a differential presence (FDR < 0.001), with a mean relative abundance of 4.18 ± 10.74%, and was detected only in the HG group. Furthermore, the Firmicutes/Bacteroidota (F/B) ratio was markedly elevated in PG (81.13) compared with HG (17.75; *p* = 0.018), highlighting a pronounced microbial imbalance in PG.

#### 3.3.2. Analysis of Gut Microbiota at the Genus Level

At the genus level, Figure 2C illustrates the 15 most abundant genera across all samples, with taxa representing <1% mean relative abundance or remaining unclassified grouped as “Others”. Among the dominant genera, *Enterococcus* and *Streptococcus* were notably enriched in PG, with mean relative abundances of 39.85 ± 42.97% and 25.69 ± 32.71%, respectively, considerably higher than those in HG (3.69 ± 3.97% and 6.24 ± 5.90%). However, individual-level analysis within PG revealed substantial heterogeneity (Figure 2D). *Enterococcus* dominated (>95%) in several samples (PG03, PG09, PG22, PG20, PG02, and PG26), while being nearly absent (<0.1%) in others (PG01, PG06, PG07, PG19, PG30 and PG17). Similarly, *Streptococcus* exceeded 80% in several samples (PG07, PG17, PG08, and PG13) but remained <0.5% in PG03, PG18, PG22, and PG02. Despite these large differences in mean abundance, Wilcoxon rank-sum tests with Benjamini–Hochberg adjustment indicated no differences between groups (FDR > 0.05), suggesting that these disparities were driven by a limited number of individuals rather than representing consistent group-level trends. In contrast, *Bifidobacterium* exhibited a higher mean relative abundance in HG (53.9 ± 28.4%) compared to PG (14.7 ± 30.0%; FDR = 0.003).

**Figure 3:**
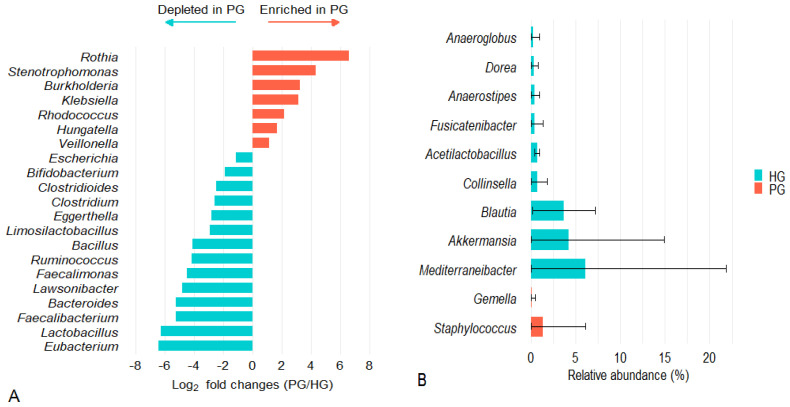
Differential abundance and exclusive presence of bacterial genera between PG and HG. (**A**) Log_2_ fold changes (PG/HG) of bacterial genera showing differences between groups (FDR < 0.05; |log_2_FC| > 1). (**B**) Genera detected exclusively in one group, indicating differences in presence/absence between PG and HG (presence in ≥50% of samples; mean relative abundance > 0.1%; FDR < 0.05).

A total of 21 genera shared between PG and HG exhibited differences in relative abundance (|log_2_FC| > 1; FDR < 0.05) (Figure 3A). Several health-beneficial genera were markedly depleted in PG, including *Eubacterium* (log_2_FC ≈ −6.4), *Lactobacillus* (−6.3), *Faecalibacterium* (−5.2), *Bacteroides* (−5.2), *Bacillus* (−4.1), and *Bifidobacterium* (−1.9). Conversely, several opportunistic genera were enriched in PG, most notably *Rothia* (+6.6), followed by *Stenotrophomonas* (+4.4), *Burkholderia* (+3.2), and *Klebsiella* (+3.1). Interestingly, three genera commonly regarded as potential pathogens- *Clostridium*, *Clostridioides*, and *Escherichia*-were less abundant in PG than in HG, with log_2_FC values of −2.58, −2.47, and −1.09, respectively (all FDR < 0.05).

Figure 3B illustrates genera detected exclusively in one group. In the PG, *Staphylococcus* was the notable exclusive genus, detected in 83.3% of samples with a mean relative abundance of 1.31 ± 4.79%. In contrast, nine genera were exclusive to the HG, including *Mediterraneibacter* (present in 80.0% of samples; 6.07 ± 15.81%), *Akkermansia* (60.0%; 4.18 ± 10.74%), and *Blautia* (100.0%; 3.68 ± 3.49%).

#### 3.3.3. Analysis of Gut Microbiota at the Species Level

**Figure 4:**
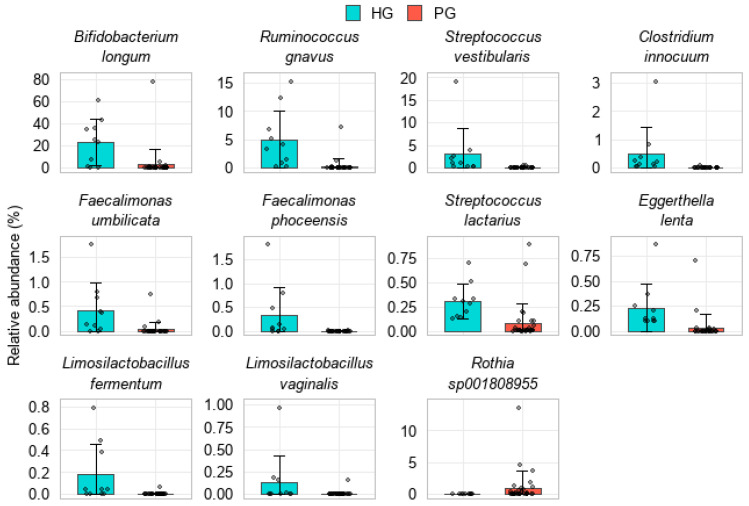
Species detected in both HG and PG showing differences in relative abundance (FDR < 0.05 and |log_2_FC| > 1).

Analysis at the species level identified 11 shared species that exhibited differences in relative abundance between PG and HG (FDR < 0.05; |log_2_FC| > 1). Several beneficial species were markedly depleted in the PG, including *Bifidobacterium longum* (22.95 ± 20.74% in HG vs. 2.86 ± 14.19% in PG; log_2_FC = −3.01), *Faecalimonas umbilicata*, *F. phoceensis*, and *Limosilactobacillus fermentum*. Other commensal species, such as *Ruminococcus gnavus*, *Streptococcus vestibularis*, *S. lactarius*, *Clostridium innocuum*, and *Eggerthella lenta*, were also reduced in the PG. In contrast, only one species, *Rothia* sp001808955, was enriched in PG (0.99 ± 2.60% vs. 0.01 ± 0.01% in HG; log_2_FC = +6.59) (Figure 4).

Table 2 shows that 3 species were exclusively detected in all PG samples, with the highest mean relative abundances observed for *Enterococcus lactis* (34.94 ± 43.81%), *Streptococcus thermophilus* (5.28 ± 9.64%), and *Streptococcus parasanguinis* (3.70 ± 7.16%).

In contrast, several species were exclusively detected in the HG group, with the 4 most abundant being *Bifidobacterium pseudocatenulatum* (12.59 ± 21.64%), *Mediterraneibacter faecis* (6.06 ± 15.81%), *Akkermansia muciniphila* (4.18 ± 10.74%), and *B. dentium* (3.68 ± 11.20%). Additionally, several other beneficial species, including *Lactobacillus johnsonii*, *Eubacterium callanderi*, *Collinsella aerofaciens*, *Limosilactobacillus mucosae*, *Fusicatenibacter saccharivorans*, and *Anaerostipes hadrus*, were present in ≥50% of HG samples, with mean relative abundances ranging from 0.3 to 1.0% (Table 3).

#### 3.3.4. Analysis of Predicted Functional Profiles of the Gut Microbiota

**Figure 5:**
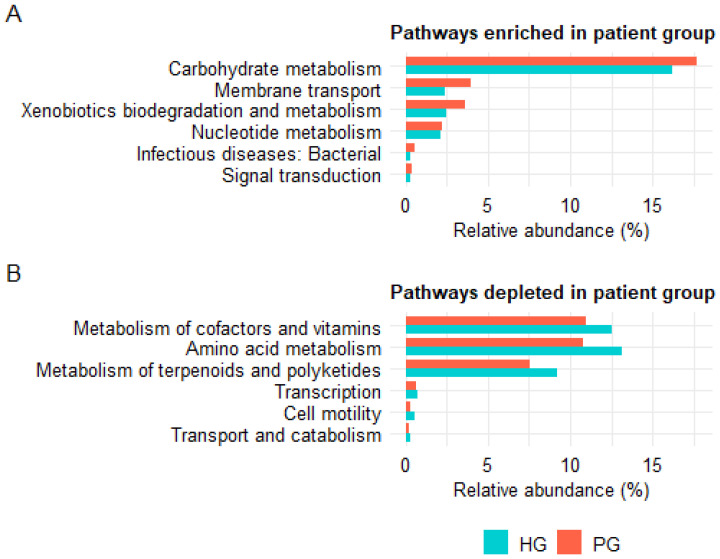
KEGG level 2 functional pathways showing differences between PG and HG, as predicted from 16S rRNA gene profiles using PICRUSt2. (**A**) Pathways enriched in PG compared to HG. (**B**) Pathways depleted in PG compared to HG. Only pathways with FDR < 0.05 and mean relative abundance > 0.1% are shown.

Functional profiling of the gut microbiota based on 16S rRNA gene data using PICRUSt2 revealed differences between PG and HG at both KEGG level 2 and level 3. At KEGG level 2, 12 functional pathways showed differences between the two groups (FDR < 0.05; mean relative abundance > 0.1%). Among them, six pathways were enriched in the PG, including three pathways classified under Metabolism (carbohydrate metabolism, xenobiotics biodegradation and metabolism, nucleotide metabolism), two under Environmental Information Processing (membrane transport, signal transduction), and one under Human Diseases (infectious diseases: bacterial) (Figure 5A). Conversely, six pathways were depleted in the PG, including three within Metabolism (amino acid metabolism, metabolism of cofactors and vitamins, metabolism of terpenoids and polyketides), one within Cellular Processes (transport and catabolism), and two pathways associated with Genetic Information Processing (transcription) and Cellular Processes (cell motility) (Figure 5B).

**Figure 6:**
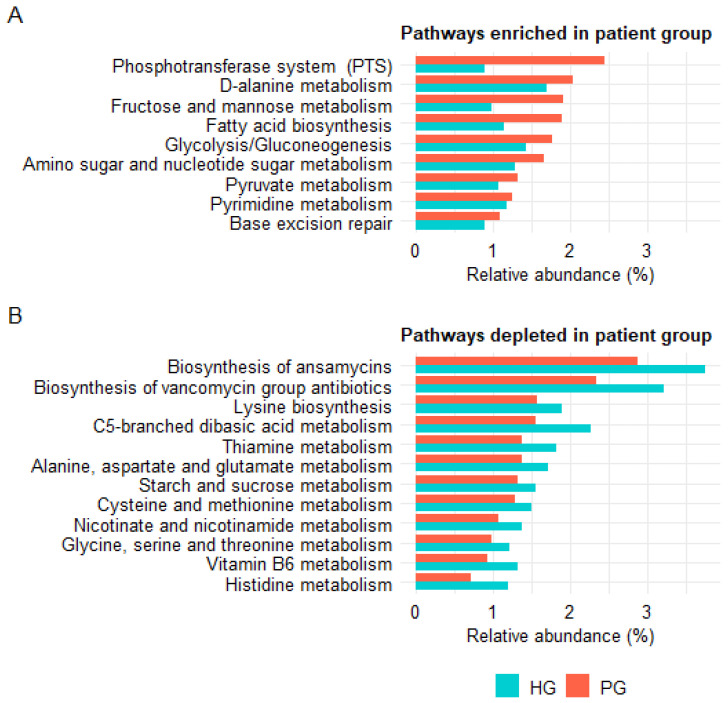
KEGG level 3 functional pathways significantly differed between PG and HG, as predicted from 16S rRNA gene profiles using PICRUSt2. (**A**) Pathways significantly enriched in PG relative to HG. (**B**) Pathways significantly depleted in PG relative to HG. Only pathways with FDR < 0.05 and relative abundance > 1% are shown.

At KEGG level 3, 21 functional pathways showed differences between PG and HG (FDR < 0.05; mean relative abundance > 1%). Among these, nine pathways were enriched in the PG, including those involved in carbohydrate metabolism (fructose and mannose metabolism, glycolysis/gluconeogenesis, pyruvate metabolism), membrane transport (phosphotransferase system (PTS)), glycan biosynthesis and metabolism (amino sugar and nucleotide sugar metabolism), lipid metabolism (fatty acid biosynthesis), nucleotide metabolism (pyrimidine metabolism), amino acid metabolism (D-alanine metabolism), and genetic information processing (base excision repair) (Figure 6A). In contrast, twelve pathways were depleted in PG, including pathways related to amino acid metabolism (histidine metabolism, lysine biosynthesis, cysteine and methionine metabolism, alanine, aspartate and glutamate metabolism, glycine, serine and threonine metabolism, C5-branched dibasic acid metabolism), B-vitamin metabolism (thiamine metabolism, vitamin B6 metabolism, nicotinate and nicotinamide metabolism), and carbohydrate metabolism (starch and sucrose metabolism). Notably, two pathways involved in the biosynthesis of microbial-derived natural antibiotics, namely biosynthesis of ansamycins and biosynthesis of vancomycin group antibiotics, were also reduced in the PG; both belong to the KEGG category of secondary metabolite biosynthesis (Figure 6B).

## 4. Discussion

### 4.1. Alterations in Alpha Diversity

Alpha diversity indices are commonly applied in gut microbiota research to characterize species richness, evenness, and overall community diversity. The Shannon index integrates both richness and evenness, with higher values indicating a more diverse and balanced microbial ecosystem [29,30]. In contrast, the Simpson index emphasizes dominance, where higher values indicate that the community is skewed toward a few predominant species, reflecting reduced diversity [31,32].

In the present study, the PG exhibited lower microbial diversity and an altered community structure, as evidenced by a reduction in the Shannon index and an increase in the Simpson index compared with the HG. Although richness-oriented metrics such as Chao1, ACE, and the observed species count did not show marked differences, the notable reduction in the Shannon index, accompanied by elevated Simpson index values, suggests a trend toward a loss of diversity and evenness—features characteristic of gut dysbiosis.

These observations are consistent with previous reports. H.C. The et al. reported a significantly lower Shannon index in Vietnamese children with diarrhea, indicating reduced gut microbial diversity [33]. Similarly, a large-scale study conducted by Tesfaw et al. in Ethiopia, involving over 600 pediatric subjects, also observed a decrease in Shannon diversity in diarrheal cases, reinforcing that gut microbial imbalance is a common hallmark of pediatric diarrheal diseases [20].

This reduction has important clinical implications, as microbial diversity plays a protective role against pathogens through competition for nutrients and ecological niches. In a diverse microbiota, multiple species utilize the same nutrient sources required by pathogens, thereby limiting their growth and colonization [34]. Moreover, a diverse microbial community helps maintain intestinal homeostasis and produces protective metabolites, such as short-chain fatty acids (SCFAs), which exert inhibitory effects on pathogenic bacteria [35]. Therefore, the observed loss of microbial diversity may represent both a consequence of diarrhea and a potential contributing factor to its persistence and severity.

### 4.2. Gut Microbiota Dysbiosis and the Firmicutes/Bacteroidota Ratio

Gut microbiota analysis revealed a pronounced increase in the phylum Firmicutes in the PG. In contrast, the relative abundances of Actinobacteriota, Bacteroidota, and Verrucomicrobiota were notably decreased compared with the HG. These alterations led to a markedly elevated F/B ratio, a well-established indicator of gut microbial balance [36,37]. In our study, the F/B ratio in the PG reached 81.1, far exceeding the regional average of approximately 2.24 reported among healthy Asian children [38]. The increased abundance of Firmicutes in the digestive tract may explain the enhancement of Firmicutes genes in the intestinal phage community of the PG, as demonstrated by Dao et al. [27], wherein the F/B ratio reached 1.75. This unusually high F/B ratio may indicate a state of pronounced dysbiosis in the gut microbiota of children with persistent diarrhea.

The phyla Firmicutes and Bacteroidota are the predominant constituents of a healthy gut microbiota. Firmicutes comprise Gram-positive bacteria mainly belonging to the genera *Bacillus*, *Clostridium*, *Enterococcus*, *Lactobacillus*, and *Ruminococcus*, whereas Bacteroidota include beneficial Gram-negative anaerobes such as *Bacteroides*, *Alistipes*, *Parabacteroides*, and *Prevotella* [37]. The marked increase in Firmicutes, accompanied by the depletion of Bacteroidota in children with persistent diarrhea, suggests a shift toward Gram-positive dominance and the loss of beneficial Gram-negative taxa, reflecting a dysbiotic gut microbiota. This imbalance may alter metabolic and immune-regulatory processes, potentially leading to increased intestinal permeability, disruption of the mucosal barrier, and persistence of intestinal inflammation [39].

### 4.3. Deficiency of SCFA-Producing and Beneficial Bacteria

Although Firmicutes accounted for approximately 79.06% of the bacterial community in the PG, several key beneficial genera within this phylum were depleted, as also observed in the phage’s associated bacterial genes in a previous study [27]. Genera that were reduced in the PG compared with the HG included *Eubacterium*, *Lactobacillus*, *Faecalibacterium* (*F. phoceensis* and *F. umbilicata*), *Lawsonibacter*, *Faecalimonas*, *Ruminococcus*, *Bacillus*, and *Limosilactobacillus* (*L. fermentum*, *L. vaginalis*). The genes of *Bifidobacterium, Moraxella*, *Bacteroides*, *Faecalibacterium* and *Lactobacillus* in the phageome data indicated a reduced abundance in the PG [27]. Among these, *Faecalibacterium*, *Eubacterium*, and *Ruminococcus* are recognized as major producers of SCFAs, particularly butyrate, acetate, and propionate. SCFAs play vital roles in nourishing intestinal epithelial cells, maintaining mucosal barrier integrity, suppressing inflammation, and regulating local immune homeostasis [40,41,42]. In addition, *Lactobacillus*, *Bacillus*, and *Limosilactobacillus* are widely regarded as probiotic taxa that support epithelial defenses and immune modulation [43,44,45,46]. The results of our study showed that the depletion of these organisms in the PG may indicate a loss of critical functional capacities within the gut microbiota. These findings are consistent with those of Tesfaw et al., who also reported a significant depletion of commensal bacteria in patients with diarrhea, particularly in persistent cases [20].

In the HG, the proportion of Firmicutes was lower than in the PG (36.47% vs. 79.06%). However, the Firmicutes community in the HG was enriched with beneficial genera and species that are well recognized for their physiological contributions to gut homeostasis. Notably, the genera *Fusicatenibacter* (*F. saccharivorans*), *Anaerostipes* (*A. hadrus*), *Blautia* (*B. luti*, *B. argi*), and *Acetilactobacillus* (*A. jinshanensis*) were exclusively detected in the HG. Consistently, abundant *Blautia*-associated genes were detected in HG but were absent in PG in the phageome data [27]. Among these, *Anaerostipes* and *Blautia* are known SCFA producers, particularly butyrate, which is essential for maintaining the intestinal epithelial barrier and modulating immune responses [40,41]. *Lactobacillus johnsonii*, *Lacticaseibacillus paracasei*, and *Limosilactobacillus mucosae* were also exclusively found in the HG and were completely absent in the PG. These lactic acid–producing bacteria play important roles in intestinal barrier protection and immune modulation and have been studied as promising probiotics for managing intestinal inflammation [47,48,49]. The complete absence of these protective taxa in the PG may reflects a quantitative loss of beneficial microbes and a profound depletion of functionally important members of the gut ecosystem. This disruption may weaken the mucosal barrier, promote chronic inflammation, increase intestinal permeability, and impair epithelial regeneration in children with persistent diarrhea [40,50].

In contrast to the expansion of Firmicutes, the phyla Actinobacteriota and Bacteroidota showed a marked decline in children with persistent diarrhea. The relative abundances of several beneficial genera and species, including *Bifidobacterium* (*B. longum*), *Eggerthella* (*E. lenta*), and *Bacteroides*, were reduced in the PG compared with the HG. Notably, *Bifidobacterium* and *Bacteroides* are widely recognized as core members of a healthy gut microbiota. These bacteria ferment dietary fibers to produce SCFAs, modulate intestinal pH, enhance mucosal immunity, suppress pathogens, and maintain epithelial barrier integrity [51,52,53]. Despite their well-established physiological functions, *B. pseudocatenulatum*, *B. bifidum*, and *B. dentium* were not detected in the PG. *B. pseudocatenulatum* and *B. bifidum* are known for fermenting oligosaccharides and producing SCFAs such as acetate and lactate, thereby stabilizing colonic pH and inhibiting the growth of pathogens [54,55]. In parallel, *B. dentium* plays a role in mucin degradation and supports the integrity of the intestinal mucus layer, a critical line of defense for the epithelium [56]. These findings are consistent with those of Monira et al., who reported that strict anaerobes, including *Bacteroides*, *Clostridium*, *Bifidobacterium*, *Lactobacillus*, and *Eubacterium*, were present at levels three- to fourfold lower in individuals with diarrhea compared with healthy controls [57].

*Akkermansia muciniphila*, an obligate anaerobe belonging to the phylum Verrucomicrobiota, plays a crucial role in maintaining the integrity of the intestinal mucus barrier. *A. muciniphila* utilizes mucin, primarily MUC2, as its main energy source, contributing to the degradation and continuous renewal of the mucus layer. This process stimulates epithelial cells to secrete additional mucin, thereby preserving mucosal thickness and promoting tissue repair [58,59,60]. In our study, *A. muciniphila* was exclusively detected in the HG and was completely absent in the PG. In agreement, *A. muciniphila*-associated genes were abundant in the HG group but absent in the PG group according to the phageome data [27]. The absence of this mucin-degrading and mucin-restoring species may lead to thinning of the protective mucus layer, increased intestinal permeability, diminished clearance of bacteria and antigens, sustained inflammation, and impaired nutrient absorption. Furthermore, disruption of mucin homeostasis may compromise lubrication and hydration of the mucosal surface, thereby exacerbating diarrhea through epithelial damage, excessive secretion, and abnormal gut motility [59,61].

The genera *Clostridium*, *Clostridioides*, and *Escherichia* are commonly known to include pathogenic species associated with diarrhea, such as *Clostridium difficile*, *Clostridium perfringens*, and diarrheagenic *Escherichia coli* strains (for example, ETEC, EHEC, and EIEC). However, in this study, the relative abundances of these three genera were higher in the HG compared with the PG. Although this may initially appear paradoxical, it aligns with current understanding of the functional diversity within the gut microbiota. In healthy individuals, many species within *Clostridium* and *Clostridioides* are beneficial obligate anaerobes that produce butyrate, a key SCFA essential for nourishing intestinal epithelial cells, maintaining mucosal barrier integrity, and regulating mucosal immune responses [62]. Likewise, most *E. coli* strains found in the healthy gut are commensals that help sustain the anaerobic environment, synthesize vitamins, support epithelial homeostasis, and stimulate mucosal immunity. In addition, commensal *E. coli* strains can suppress pathogens by producing bacteriocins, competing for nutrients, and occupying ecological niches [63]. Therefore, the higher abundance of *Clostridium*, *Clostridioides*, and *Escherichia* in the HG is more likely indicative of the beneficial roles of their commensal members rather than representing evidence of pathological colonization.

### 4.4. Expansion of Opportunistic and Pathogenic Taxa in the PG

Alongside the substantial depletion of beneficial obligate anaerobes, the PG exhibited a marked increase in facultative anaerobic and aerobic bacteria with potential pathogenicity. These included *Streptococcus*, *Enterococcus*, *Staphylococcus*, *Rhodococcus*, *Rothia*, *Stenotrophomonas*, *Burkholderia*, *Klebsiella*, and *Gemella*. Particularly, species such as *Streptococcus thermophilus*, *S. parasanguinis*, *S. mitis*, *Enterococcus lactis*, *Staphylococcus aureus*, *Escherichia ruysiae*, and *Klebsiella oxytoca* were exclusively detected in the PG, suggesting a possible link to gut dysbiosis and a potential role in the pathogenesis of persistent diarrhea. These results are consistent with previous findings that diarrheal samples (PG07, PG09, PG17, PG24, PG26, PG02, PG03, PG05, and PG25, classified within clusters PV3–PV5; and PG20, PG22, and PG23, clustered in PV6) contained more than 90% bacterial genes derived from *Enterococcus*. In addition, *Klebsiella*, *Veillonella*, *Staphylococcus*, *Haemophilus*, *Mordavella*, *Salmonella* and *Escherichia* were also observed at high abundance in the PG [27].

This microbial alteration is consistent with findings of Quaye et al. [64], who demonstrated that the gut microbiota of patients with acute diarrhea was enriched with opportunistic genera, including *Enterococcus*, *Streptococcus*, and *Staphylococcus*. In contrast, healthy controls showed enrichment of beneficial genera such as *Faecalibacterium*, *Prevotella*, *Ruminococcus*, and *Bacteroides* [64]. Similarly, Fan et al. observed a shift in microbial composition from health-associated genera, such as *Lactobacillus* and *Bifidobacterium*, in healthy children to potentially pathogenic genera, including *Klebsiella* and *Streptococcus*, in children with diarrhea [65].

A study by H.C. The et al. on children with infectious diarrhea in Vietnam also reported a profound loss of SCFA-producing obligate anaerobes and a significant enrichment of aerobic and facultative anaerobic species [33]. These changes align with previously described mechanisms in which intestinal inflammation and antibiotic exposure contribute to dysbiosis by reducing microbial diversity and favoring the expansion of opportunistic bacteria such as Enterococci, particularly antibiotic-resistant cocci [66,67,68,69,70]. In our study, 86.7% of children in the PG had received antibiotics, which may have contributed to the expansion of opportunistic taxa such as *Enterococcus*, *Streptococcus*, and *Staphylococcus*. Moreover, post-diarrheal malabsorption may lead to excess luminal monosaccharides and proteins, providing substrates that promote the proliferation of cocci [71]. Inflammation-induced mucosal barrier disruption may also increase oxygen diffusion into the gut lumen. This environment is typically anaerobic, thereby inhibiting the growth of obligate anaerobes and promoting the growth of aerotolerant and facultative anaerobic taxa [72,73,74]. Therefore, the enrichment of genera such as *Streptococcus*, *Enterococcus*, *Klebsiella*, and *Staphylococcus* in PG may be associated with mucosal injury and local inflammation, which can increase luminal oxygen tension. Together with the effects of antibiotic treatment, these factors may create a favorable environment for the expansion of aerobic and facultative anaerobic bacteria, which can outcompete and partially replace the beneficial obligate anaerobes that have been depleted [72,73,75].

*Rothia* and *Gemella* are core members of the oral microbiota in healthy individuals, where they act as commensals but may also cause opportunistic infections [76,77]. In our study, *Rothia* exhibited a higher mean relative abundance in the PG, while *Gemella* was exclusively detected in this group. These findings are consistent with a previous study from Vietnam that investigated early-stage gut dysbiosis in children with infectious diarrhea and reported the abnormal presence of oral-origin bacteria such as *Rothia* and *Gemella* in fecal samples. Notably, *Rothia* was identified as one of the most abundant taxa in the stool of children with infectious diarrhea [33]. Several international studies have also reported an increase in *Rothia* during diarrheal episodes, suggesting the possible translocation of oral bacteria to the lower gastrointestinal tract under dysbiotic conditions [64,78].

Although not commonly recognized as major diarrheal pathogens, our study identified several potentially pathogenic bacterial species in the PG, including *Klebsiella oxytoca* and *Escherichia ruysiae*. *K. oxytoca* is a Gram-negative bacillus capable of producing cytotoxins and has been implicated in antibiotic-associated hemorrhagic colitis [79]. A large-scale study analyzing 5581 diarrheal stool samples reported that 2.1% were positive for *K. oxytoca* [80]. *Escherichia ruysiae* is a recently identified species within the *Escherichia* genus, and its pathogenic potential remains poorly understood [81]. Some strains have been found to harbor genes associated with antimicrobial resistance, raising concerns about their impact on human health [82]. Although *E. ruysiae* has been isolated from individuals with diarrhea, additional studies are needed to elucidate its specific role in disease pathogenesis [82].

### 4.5. Functional Predictions from PICRUSt2 and Their Biological Implications

In addition to compositional alterations, predicted functional changes in the gut microbiota also appear to play an important role in the pathogenesis of persistent diarrhea. Functional prediction using the PICRUSt2 tool revealed marked differences between the PG and the HG. It should be noted that the predicted functions derived from PICRUSt2 reflect inferred metabolic potential based on 16S rRNA gene profiles, not direct measurements of functional activity. The PG exhibited clear enrichment of carbohydrate metabolism pathways, including glycolysis, pyruvate metabolism, and fructose and mannose metabolism, along with higher scores for the PTS. This bacterial transport system facilitates the efficient uptake and translocation of sugars. Under diarrheal conditions, unabsorbed carbohydrates in the small intestine can reach the colon and undergo fermentation, producing organic acids and other metabolites that may increase luminal osmotic pressure and thereby potentially prolong diarrhea. Excess luminal carbohydrates may further stimulate the expression of carbohydrate-metabolizing enzymes and membrane transport systems by gut microbiota members [83,84].

Findings from this study indicate that the predicted functional profile of the gut microbiota in children with persistent diarrhea differs from patterns reported in acute diarrhea. Xiao et al. reported that membrane transport activity was reduced in children with acute rotavirus gastroenteritis compared with healthy controls [22]. Pop et al. observed that, in children with moderate-to-severe acute diarrhea, pathways characteristic of obligate anaerobes, such as glycolysis, pyruvate metabolism, short-chain fatty acid biosynthesis, and xylene degradation, were decreased, whereas oxygen-dependent pathways were increased in the diarrheal group [23]. The alteration in carbohydrate metabolism observed in the PG may reflect a potential restructuring of the gut microbial community, characterized by a reduction in obligate anaerobes such as *Bifidobacterium*, *Faecalibacterium*, and *Anaerostipes*, accompanied by an increase in aerobic or facultatively anaerobic taxa such as *Enterococcus* and *Staphylococcus*. These aerobic taxa efficiently utilize carbohydrates in oxygen-rich environments and often exhibit abundant expression of carbohydrate-metabolizing enzymes and the PTS [85,86].

This replacement may promote functional compensation, maintaining or even enhancing carbohydrate metabolic activity in a state of dysbiosis. The composition of the gut microbiota shapes the final products of metabolic pathways. Although the PG exhibited enrichment of carbohydrate metabolism pathways such as glycolysis and pyruvate metabolism, this does not necessarily indicate increased SCFA synthesis. Pyruvate, the principal intermediate of carbohydrate catabolism, can be converted into lactate, succinate, or acetyl-CoA. To generate SCFAs, these intermediates must undergo further transformation by cross-feeding bacteria, including *Faecalibacterium*, *Anaerostipes*, *Eubacterium*, *Bacteroides*, and *Bifidobacterium* [87,88,89]. In our study, all of these genera were reduced in the PG, which may have led to incomplete fermentation and decreased production of beneficial SCFAs. Conversely, the expansion of aerobic or facultatively anaerobic bacteria may facilitate the accumulation of fermentation products such as lactate or succinate, altering luminal pH and exerting potentially detrimental effects on the gut environment [5]. Thus, enrichment of carbohydrate metabolism pathways in the PG does not reflect a healthy microbiota but instead suggests a restructured microbial community adapted to chronic inflammation, excess unabsorbed carbohydrates, and a lack of taxa with optimal metabolic functions for the host. Such changes may contribute to the persistence and prolongation of diarrhea in children.

Conversely, the PG exhibited a marked reduction in amino acid, cofactor, and vitamin metabolism pathways. The decreased activity of amino acid metabolism may reflect a deficiency of beneficial bacteria involved in the synthesis or degradation of amino acids, potentially impairing nutrient absorption and contributing to the malnutrition commonly observed in children with persistent diarrhea [90]. Notably, the decline in pathways associated with B-vitamin metabolism, including thiamine, vitamin B6, nicotinate, and nicotinamide, may have adverse consequences. The gut microbiota plays a critical role in synthesizing these vitamins, which are essential cofactors in numerous metabolic reactions of bacteria and the host. B-vitamin deficiencies may impair intestinal barrier function, immune regulation, and systemic energy metabolism [91,92].

A noteworthy observation in this study was the predicted reduction in secondary metabolite biosynthesis pathways, particularly biosynthesis of ansamycins and biosynthesis of vancomycin group antibiotics. These pathways are associated with the endogenous antimicrobial capacity of the gut microbiota, contributing to ecosystem balance and suppression of enteric pathogens [93]. This finding aligns with the study by Dinleyici et al., which reported decreased expression of the biosynthesis of the ansamycins pathway in children with acute rotavirus gastroenteritis and proposed that this functional deficiency may be linked to impaired mucosal protection in the context of gastrointestinal infections [94].

### 4.6. Study Limitations and Future Research Directions

One limitation of this study is that the 30 stool samples from the HG were pooled into 10 composite samples prior to sequencing. Although this strategy reduced sequencing costs, it also diminished data resolution, thereby limiting the assessment of inter-individual variability and the ability to control for confounding factors such as age, sex, and diet. Furthermore, the mean age of the PG was significantly lower than that of the HG, which may have introduced an additional confounder in the gut microbiota analyses. Consequently, some of the observed differences between groups may reflect age-related or individual-level variation rather than disease status alone.

Another important limitation concerns antibiotic exposure. None of the healthy children had received antibiotics for at least one month, whereas 86.7% of PG participants had a history of antibiotic use prior to sampling. This high prevalence of antibiotic exposure likely contributed to some of the observed dysbiosis and should be taken into account when interpreting the findings. Moreover, the inclusion criterion for healthy controls required at least one month without antibiotic use. Although this interval may not completely eliminate antibiotic-induced alterations in the gut microbiota, it was chosen based on practical considerations in Vietnam, where non-prescription antibiotic use is common [95,96], and young children (6–24 months) are frequently exposed to respiratory and ear–nose–throat infections that often require antibiotics. Additionally, culture for some enteric pathogens, such as *Yersinia enterocolitica* and *Campylobacter* spp., was not performed, which represents a diagnostic limitation.

Future studies should include larger sample sizes, with individual-level sequencing data and more detailed clinical metadata, to enable statistical adjustment for confounding factors such as age, diet, and antibiotic exposure.

## 5. Conclusions

This study demonstrates that children with persistent diarrhea of unknown etiology exhibit marked gut microbiota dysbiosis, characterized by the depletion of commensal SCFA- and lactic acid-producing taxa such as *Bifidobacterium longum*, *Faecalibacterium*, *Lactobacillus*, and *Eubacterium*, alongside the enrichment of opportunistic organisms including *Klebsiella*, *Enterococcus lactis*, and *Streptococcus* spp. Functional predictions using PICRUSt2 indicated increased carbohydrate metabolism and membrane transport but reductions in amino acid metabolism, B-vitamin biosynthesis, and endogenous antimicrobial pathways. These alterations may not merely reflect a secondary consequence but could be associated with processes that promote inflammation and compromise mucosal barrier function. Overall, our findings provide further evidence that gut microbial imbalance may be associated with the pathogenesis of persistent diarrhea in early childhood. Microbiota-targeted interventions, including probiotics, prebiotics, dietary modification, and fecal microbiota transplantation, warrant further investigation as potential approaches to improve clinical outcomes in affected children.

## Figures and Tables

**Table 1:** Baseline characteristics of the study participants (*n* = 60).

Characteristics		HG (*n* = 30)	PG (*n* = 30)	*p*-Value
Age, months (Mean ± SD)		11.4 ± 3.8	7.2 ± 2.0	<0.001 ^a^
Age group, *n* (%)	6–12 months	19 (63.3%)	29 (96.7%)	0.002 ^b^
	13–24 months	11 (36.7%)	1 (3.3%)	
Sex, *n* (%)	Male	16 (53.3%)	18 (60.0%)	0.602 ^c^
	Female	14 (46.7%)	12 (40.0%)	
Residence, *n* (%)	Rural	12 (40.0%)	19 (63.3%)	0.070 ^c^
	Urban	18 (60.0%)	11 (36.7%)	
Mode of delivery, *n* (%)	Vaginal	16 (53.3%)	11 (36.7%)	0.195 ^c^
	Cesarean	14 (46.7%)	19 (63.3%)	
Feeding type (0–4 months)	Exclusive breastfeeding	11 (36.7%)	11 (36.7%)	1.000 ^b^
	Mixed feeding	15 (50.0%)	16 (53.3%)	
	Exclusive formula feeding	4 (13.3%)	3 (10.0%)	
Timing of complementary feeding	Not yet introduced	1 (3.3%)	7 (23.3%)	0.073 ^b^
	Introduced at 4–6 months	14 (46.7%)	13 (43.3%)	
	Introduced after 6 months	15 (50.0%)	10 (33.4%)	
Drinking water source	Tap water	22 (73.3%)	21 (70.0%)	0.775 ^c^
	Other (well, rain, river)	8 (26.7%)	9 (30.0%)	
Number of children	≤2 children	22 (73.3%)	24 (80.0%)	0.543 ^c^
	≥3 children	8 (26.7%)	6 (20.0%)	
Childcare	At home	29 (96.7%)	25 (83.3%)	0.196 ^b^
	Daycare	1 (3.3%)	5 (16.7%)	
WAZ	WAZ < −2 SD	0 (0.0%)	1 (3.3%)	1.000 ^b^
	−2 SD ≤ WAZ ≤ 2 SD	29 (96.7%)	29 (96.7%)	
	WAZ > 2 SD	1 (3.3%)	0 (0.0%)	
HAZ	HAZ < −2 SD	2 (6.7%)	4 (13.3%)	0.748 ^b^
	−2 SD ≤ HAZ ≤ 2 SD	26 (86.6%)	25 (83.4%)	
	HAZ > 2 SD	2 (6.7%)	1 (3.3%)	
Antibiotic use in the past 30 days, *n* (%)		0 (0%)	26 (86.7%)	-

Values are presented as Mean ± SD or *n* (%). *p*-values were calculated using ^a^ Wilcoxon rank-sum test, ^b^ Fisher’s exact test, or ^c^ Chi-square test, as appropriate. WAZ: Weight-for-Age Z-score; HAZ: Height-for-Age Z-score.

**Table 2:** Bacterial species exclusively detected in the patient group with FDR < 0.05, mean relative abundance > 0.1%, and prevalence ≥ 50% of samples.

Species	Mean PG	SD PG	FDR	Patient Positive *n* (%)
*Enterococcus lactis*	34.94	43.81	<0.0001	30 (100)
*Streptococcus thermophilus*	5.28	9.64	<0.0001	30 (100)
*Streptococcus parasanguinis*	3.70	7.16	<0.0001	30 (100)
*Staphylococcus aureus*	1.31	4.79	<0.0001	25 (83.3)
*Lacticaseibacillus rhamnosus*	1.28	4.76	0.002	19 (63.3)
*Klebsiella oxytoca*	1.04	4.38	0.004	18 (60.0)
*Escherichia ruysiae*	0.87	1.94	<0.0001	29 (96.7)
*Streptococcus mitis*	0.15	0.25	<0.0001	27 (90.0)

**Table 3:** Bacterial species exclusively detected in the healthy group with FDR < 0.05, mean relative abundance > 0.1%, and prevalence ≥ 50% of samples.

Species	Mean HG	SD HG	FDR	Healthy Positive *n* (%)
*Bifidobacterium pseudocatenulatum*	12.59	21.64	<0.0001	7 (70)
*Mediterraneibacter faecis*	6.06	15.81	<0.0001	7 (70)
*Akkermansia muciniphila*	4.18	10.74	<0.0001	6 (60)
*Bifidobacterium dentium*	3.68	11.20	<0.0001	8 (80)
*Enterococcus avium*	3.28	3.73	<0.0001	10 (100)
*Blautia luti*	1.97	2.23	<0.0001	8 (80)
*Lactobacillus johnsonii*	0.98	1.61	<0.0001	6 (60)
*Blautia argi*	0.84	0.96	<0.0001	9 (90)
*Eubacterium callanderi*	0.74	0.95	<0.0001	10 (100)
*Bifidobacterium bifidum*	0.72	0.95	0.002	5 (50)
*Acetilactobacillus jinshanensis*	0.69	0.27	<0.0001	10 (100)
*Collinsella aerofaciens*	0.66	1.08	<0.001	6 (60)
*Limosilactobacillus mucosae*	0.48	0.88	0.002	5 (50)
*Fusicatenibacter saccharivorans*	0.42	0.86	0.002	5 (50)
*Anaerostipes hadrus*	0.33	0.57	0.002	5 (50)
*Lacticaseibacillus paracasei*	0.30	0.58	0.002	5 (50)
*Anaeroglobus micronuciformis*	0.26	0.69	<0.001	7 (70)
*Klebsiella pneumoniae* 723684	0.15	0.21	<0.001	9 (90)

## Data Availability

All the metagenomic data generated from this study were enclosed in the Tables S7–S10.

## Supplementary Materials

The following supporting information can be downloaded at: https://www.mdpi.com/article/10.3390/pathogens14111136/s1, Table S1: Alpha diversity indices of gut microbiota; Table S2: Relative abundance of gut microbiota at the phylum level; Table S3: Relative abundance of gut microbiota at the genus level; Table S4: Relative abundance of gut microbiota at the species level; Table S5: KEGG level 2 functional pathways of gut microbiota predicted by PICRUSt2; Table S6: KEGG level 3 functional pathways of gut microbiota predicted by PICRUSt2; Table S7: List of OTUs and their abundance in each PG sample; Table S8: Sequences of OTUs in the diarrheal group; Table S9: List of OTUs and their abundance in each HG sample; and Table S10: Sequences of OTUs in the healthy group.

## Abbreviations

The following abbreviations are used in this manuscript:

PG	Patient Group
HG	Healthy Group
NGS	Next-generation sequencing
WHO	World Health Organization
OTUs	Operational taxonomic units
RDP	Ribosomal Database Project
KEGG	Kyoto Encyclopedia of Genes and Genomes
FDR	False discovery rate
IQR	Interquartile range
F/B ratio	Firmicutes/Bacteroidota ratio
SCFAs	Short-chain fatty acids
PTS	Phosphotransferase system
